# Repurposing approved non-oncology drugs for cancer therapy: a comprehensive review of mechanisms, efficacy, and clinical prospects

**DOI:** 10.1186/s40001-023-01275-4

**Published:** 2023-09-14

**Authors:** Roohi Mohi-ud-din, Apporva Chawla, Pooja Sharma, Prince Ahad Mir, Faheem Hyder Potoo, Željko Reiner, Ivan Reiner, Dilek Arslan Ateşşahin, Javad Sharifi-Rad, Reyaz Hassan Mir, Daniela Calina

**Affiliations:** 1 Department of General Medicine, Sher-I-Kashmir Institute of Medical Sciences (SKIMS), Srinagar, Jammu and Kashmir 190001 India; 2 Khalsa College of Pharmacy, G.T. Road, Amritsar, Punjab 143001 India; 3 Department of Pharmacology, College of Clinical Pharmacy, Imam Abdulrahman Bin Faisal University, 1982, 31441 Dammam, Saudi Arabia; 4 Department of Internal Medicine, School of Medicine, University Hospital Center Zagreb, Zagreb, Croatia; 5 Department of Nursing Sciences, Catholic University of Croatia, Ilica 242, 10000 Zagreb, Croatia; 6 Baskil Vocational School, Department of Plant and Animal Production, Fırat University, 23100 Elazıg, Turkey; 7 Facultad de Medicina, Universidad del Azuay, Cuenca, Ecuador; 8 Pharmaceutical Chemistry Division, Department of Pharmaceutical Sciences, University of Kashmir, Hazratbal, Srinagar, Kashmir 190006 India; 9 Department of Clinical Pharmacy, University of Medicine and Pharmacy of Craiova, 200349 Craiova, Romania

**Keywords:** Repurposing, Non-oncology drugs, Cancer therapy, Mechanisms, Efficacy, Clinical prospects

## Abstract

Cancer poses a significant global health challenge, with predictions of increasing prevalence in the coming years due to limited prevention, late diagnosis, and inadequate success with current therapies. In addition, the high cost of new anti-cancer drugs creates barriers in meeting the medical needs of cancer patients, especially in developing countries. The lengthy and costly process of developing novel drugs further hinders drug discovery and clinical implementation. Therefore, there has been a growing interest in repurposing approved drugs for other diseases to address the urgent need for effective cancer treatments. The aim of this comprehensive review is to provide an overview of the potential of approved non-oncology drugs as therapeutic options for cancer treatment. These drugs come from various chemotherapeutic classes, including antimalarials, antibiotics, antivirals, anti-inflammatory drugs, and antifungals, and have demonstrated significant antiproliferative, pro-apoptotic, immunomodulatory, and antimetastatic properties. A systematic review of the literature was conducted to identify relevant studies on the repurposing of approved non-oncology drugs for cancer therapy. Various electronic databases, such as PubMed, Scopus, and Google Scholar, were searched using appropriate keywords. Studies focusing on the therapeutic potential, mechanisms of action, efficacy, and clinical prospects of repurposed drugs in cancer treatment were included in the analysis. The review highlights the promising outcomes of repurposing approved non-oncology drugs for cancer therapy. Drugs belonging to different therapeutic classes have demonstrated notable antitumor effects, including inhibiting cell proliferation, promoting apoptosis, modulating the immune response, and suppressing metastasis. These findings suggest the potential of these repurposed drugs as effective therapeutic approaches in cancer treatment. Repurposing approved non-oncology drugs provides a promising strategy for addressing the urgent need for effective and accessible cancer treatments. The diverse classes of repurposed drugs, with their demonstrated antiproliferative, pro-apoptotic, immunomodulatory, and antimetastatic properties, offer new avenues for cancer therapy. Further research and clinical trials are warranted to explore the full potential of these repurposed drugs and optimize their use in treating various cancer types. Repurposing approved drugs can significantly expedite the process of identifying effective treatments and improve patient outcomes in a cost-effective manner.

## Introduction

Cancer is a serious disease that causes high mortality rates worldwide [1–3]. The occurrence of different types of cancer and particularly the possibilities of treatment are a big challenge for clinicians [4–9]. Malignant tumors such as liver cancer, breast, prostate, pancreas and colorectal cancer are generally difficult to cure at advanced phases with existing conventional treatments [10–14]. Finding new substances for the treatment of cancers was the result of technological development and innovative approaches. Surprisingly, the research on novel agents for cancer treatment lasts long and is a complicated exercise due to the various steps necessary for the isolation, synthesis, and purification of new anticancer substances [13, 15–18]. After drug discovery, synthesis and selection of appropriate formulation and preclinical pharmacology and toxicology, phase I clinical trials have to be made which begin with the administration of an investigational drug into healthy humans This phase involves the estimation of initial safety and tolerability, pharmacokinetics and assessments of pharmacodynamics. If successful, phase II is performed with the primary objective to explore therapeutic efficacy in patients. After that, for many of them, there is only a small probability to finish successfully phase III clinical trials which are designed to confirm the preliminary evidence obtained in phase II to verify that the potential drug is safe and effective for use in the intended indication and recipient patients. The amount of time and money for the production of new anti-cancer drugs is a big obstacle for the pharma industry for new drugs development, which will have proven therapeutic efficacy [19–23]. Therefore, a novel concept of the use of old FDA-approved drugs is the recent direction to help clinicians and researchers, and, of course, the patients [24]. In this strategy, the advantage is that the assessment of drug safety, such as pharmacodynamics, pharmacokinetics, toxicity, and safety profiles, has been already previously established in Phase I studies. Moreover, old approved drugs can rapidly proceed for further Phase II clinical trials. Therefore, the interest is more and more focused on this strategy because of the lower cost and less time for developing new use of old drugs [19, 25].

## Drug repurposing landscape: a brief synopsis

**Fig. 1:**
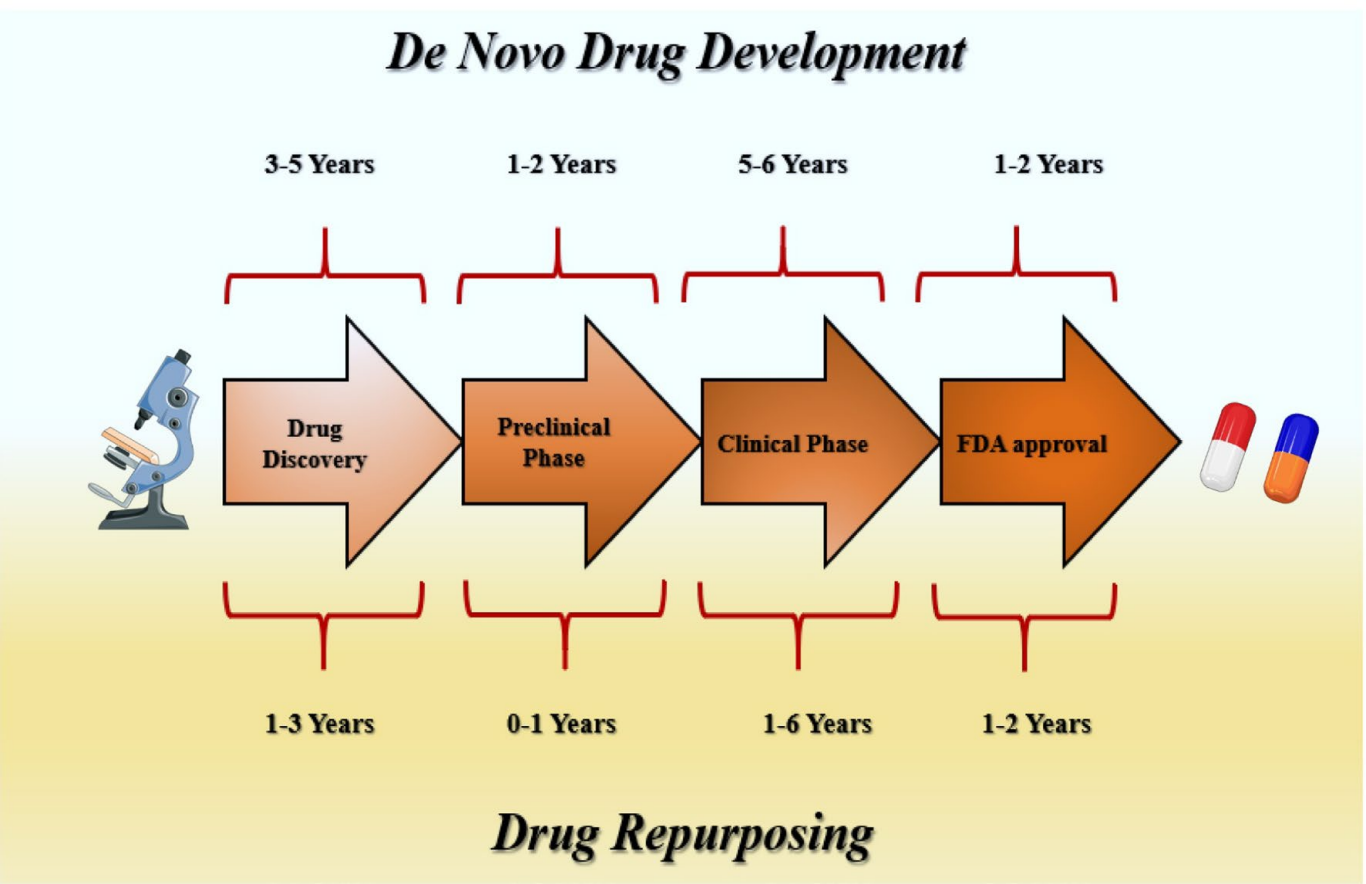
Comparison of drug repurposing with traditional drug discovery

Drug development is a multistep process that includes identifying the therapeutic drug molecule that is clinically effective in the treatment of a disease [26]. This conventional drug discovery strategy implicates the de novo identification of new molecular entities. It has five stages, such as the discovery of the molecule and preclinical study, safety review, clinical studies, FDA review, and FDA post-market safety monitoring [27]. This process involves the identification of candidate molecules, synthesis, characterization, validation, optimization, screening, and assays for therapeutic efficacy [28]. When a product shows favorable outcomes in these studies, then the molecule has to go through a drug development process and subsequently testing in clinical trials [28]. De novo drug development is a time-consuming process and involves significant investments. It usually takes years of work (10–17 years) and costs millions of dollars. Furthermore, it is associated with high failure rates, with roughly 90% of molecules being rejected due to unexpected characteristics, such as safety and efficacy concerns [29, 30]. Despite massive expenditures in drug discovery and tremendous advancements in biological and informational technology over the past several decades, the number of new drugs brought to the phase of clinical trials has not expanded so much and remained relatively constant. Even though total research and development cost for drug discovery has expanded tenfold from 1975 (US $4 billion) to 2009 ($40 billion), there has been no substantial change in the number of new molecules approved since 1975 (in 2013 there were only 6 new drug moieties approved in comparison with the year 1976, where the number of newly approved pharmaceuticals was 27) [31]. The situation has improved in the last decade but not to a large extent. The increasing cost and time in the drug discovery process have resulted in the possibility that if resistance to the available drugs emerges, people with advanced diseases will end up dying before a substitute treatment option could become accessible [32]. Drug development is undoubtedly one of the most complex tasks in pharmaceutical research. In addition to already intimidating complications in pharmacological drug design, numerous obstacles occur due to regulatory, clinical intellectual property, and economic concerns. As a result of these challenging circumstances, the drug development process has become even more prolonged and uncertain. In pursuit of new treatment alternatives for patients with diseases, such as cancer, researchers have turned to drug repurposing tactics [33]. Drug repurposing also termed as drug repositioning, drug rescuing, drug reprofiling, drug recycling, or therapeutic switching, involves identifying and exploring the new therapeutic use of already FDA-approved and in clinical practice used drugs, but used for the treatment of other indications [24]. It is regarded as the most effective strategy in developing drug candidates using novel pharmacological properties and therapeutic characteristics of well-known drugs. Considering that traditional drug discovery is a time-consuming and costly process, the revolutionary strategy of drug repositioning is used to boost the success rate of medication development. Compared to the traditional drug discovery approach, this strategy is more favorable in terms of minimizing the length of time required for drug development while maintaining low costs, high efficiency, and minimal risk of failure [34]. The drug repurposing also offers a significant advantage not only in terms of the availability of preclinical information about the existing drug (a drug to be repurposed) but also provides additional data about clinical aspects such as pharmacokinetic, pharmacodynamics, and toxicity profile of that particular drug [35]. Because of this, these drugs might quickly be tested in Phase II and Phase III clinical trials, and the accompanying development costs could be significantly lower. The risk of failure is reduced, because in-vitro and in-vivo studies, toxicology profiles, chemical optimization, and formulation development have previously been explored. As a result, pharmaceutical companies have directed more and more of their attention to drug repurposing, since it might give a considerable advantage compared to traditional drug development (Fig. 1) [36]. In this context, it is not unexpected that approximately 30% of newly approved drugs in the United States are repurposed drugs [37].

### Experimental drug repurposing approach

#### Binding assay

Techniques such as affinity chromatography, proteomics, and mass spectroscopy are used to identify novel targets for old drugs [38]. The protein target of gefitinib was investigated using HeLa cell extract. Mass spectroscopy results indicated that gefitinib could potentially interact with 20 different protein kinases that might be a target for gefitinib [27].

#### Phenotypic approaches

The phenotypic drug discovery approach is an experimental strategy that uses the library of accessible drug collections and focuses on finding their biological activities in cells and living organisms. It does not depend on the direct interaction with the target. Changes in in-vitro, in-vivo models and clinical studies can lead to the discovery of new drugs [39]. This is a screening procedure that does not presume the mechanism of action, and the primary output is a change in phenotype or physiological parameters. Cells, physiological systems, and whole organisms can all be used in this process. Depending upon the purpose or phase of drug development, each of these several systems can be used. Cell-based screening provides an increased insight into in vivo processes. On the other hand, in-vivo animal models help to assess the possible use of the existing medications for new phenotypic characteristics [40]. This method led to the discovery that astemizole and its metabolite desmethyl astemizole as effective inhibitors of *Plasmodium falciparum* growth and development [41].

#### Drug-centric approach

Drug-centric repurposing strategy is focused on forecasting new use for already approved drugs. This strategy relies heavily on substances that have the potential to interact with a wide range of targets (polypharmacological agents). Even though polypharmacological substances are responsible for triggering undesirable side effects, their activities can be used, because they offer the possibility of additional indications for a specific drug [42]. Polypharmacology seems to become the next major drug development paradigm. A considerable number of drugs are known for their ability to affect many targets simultaneously. Aspirin is used to relieve mild pain, fever and rheumatoid arthritis. It is also used as an anti-inflammatory agent in the treatment of Kawasaki disease and pericarditis. Transient ischemic attacks, ischemic stroke, myocardial infarction and even some types of cancer have all been successfully treated with this drug. Sildenafil, a phosphodiesterase inhibitor, was initially used for the management of erectile dysfunction. However, today, it is widely used for the management of pulmonary hypertension [43]. The majority of kinase inhibitors can inhibit several targets which makes them attractive options for the treatment of some types of cancer. Different multi-targeted tyrosine kinase inhibitors (TKIs) such as imatinib, nilotinib, and vandetanib were approved for clinical use in 2010 to treat solid cancers [44].

#### Target-based approach

The target-based drug repurposing approach involves the study of candidate drugs with biological targets such as receptors and proteins to different physiological responses to them. According to this method, new indications were discovered by relating a certain drug to a specific disease depending on the protein which it might target [42]. Proteins have many pathophysiological roles both in diseases and in healthy humans; dysfunctional, mutated, or misfolded proteins may trigger pathological responses that cause the development of a disease. The studies of proteins or biomarkers implicated in pathophysiological processes focus on target-based drug repurposing strategy [45]. The target-based strategy involves in silico or virtual high-throughput screening of drugs from various drug libraries or substances databases such as ligand-based screening or molecular docking proceeded by in vitro and in vivo high throughput or high content screening of drugs against a specific protein or biomarker of interest [27]. For instance, a new pharmaceutical molecule N-myristoyl transferase was discovered by this target-based approach for the treatment of filarial nematodes [46].

#### Knowledge-based

In this drug repurposing approach, models are developed that incorporate drug-related information, such as drug targets, chemical structures, route information, adverse effects, etc. These models are then used to anticipate unknown targets, biomarkers, or disease mechanisms [35].

#### Pathway or network-based

Pathway-based drug repurposing approach uses information about metabolic pathways, signaling pathways, and protein interaction networks to anticipate the similarity or relationship between a certain drug and a disease [35]. Network or pathway-based drug repurposing uses omics data to understand how drugs interact and communicate with disease targets. As a result, a specialized network with a few targets can be found in a vast network of pathways. Network-based strategies for drug repurposing are more and more in the focus of interest. Network-based computational biology focused on biomolecular interactions and omics data integration seem to be very promising. New drug repurposing research has discovered previously unknown signaling pathways in breast carcinoma subtypes [47, 48]. Kotelnikova et al. described a novel computational algorithm for the treatment planning of glioblastoma. The study analyzed gene expression data using a proprietary algorithm designed for pathway studio called sub-network enrichment analysis (SNEA). This method led to the discovery and FDA approval of fulvestrant (Faslodex1), a drug used to treat hormone receptor-positive metastatic breast carcinoma [49]. A study by Yu et al. suggested an approach for predicting potential drug–disease interactions that may be used for drugs or diseases that have or do not have associated genes. The adverse effects of drugs and disease symptoms were associated with identify drug–module and disease–module pairs using this strategy [50].

## Repurposing of non-oncology drugs to treat cancer

Repurposing non-oncology drugs to use against cancer cells is an alternative approach to provide better mitigation possibilities for people with cancer at a lower cost and more quickly. Many methods were used to explore the probable anticancer function of non-cancerous drugs. For drug repurposing of non-oncology medications many in vivo and in vitro trials on pharmacological models and cancer cell lines were performed. Numerous wide-ranging electronic databases, such as the National Institute of Health (NIH) and Molecular Libraries Initiative [51], in which the chemical substances, biological evaluation assays, and genetic relevance of the active chemical substances were used to analyze them as a tool for utilization of drugs repositioning [52]. Repurposing of non-oncology drugs works through many mechanisms, such as cancer monotherapy, inhibiting proliferative signaling, inducing cell death, regulation of cellular metabolism, activation of antitumor immunity, drug combinatorial therapy, reactivating growth suppressors, interfering with replication, decreasing angiogenesis, suppression of invasion, and metastasis [32].

### Anthelmintic drugs

**Fig. 2:**
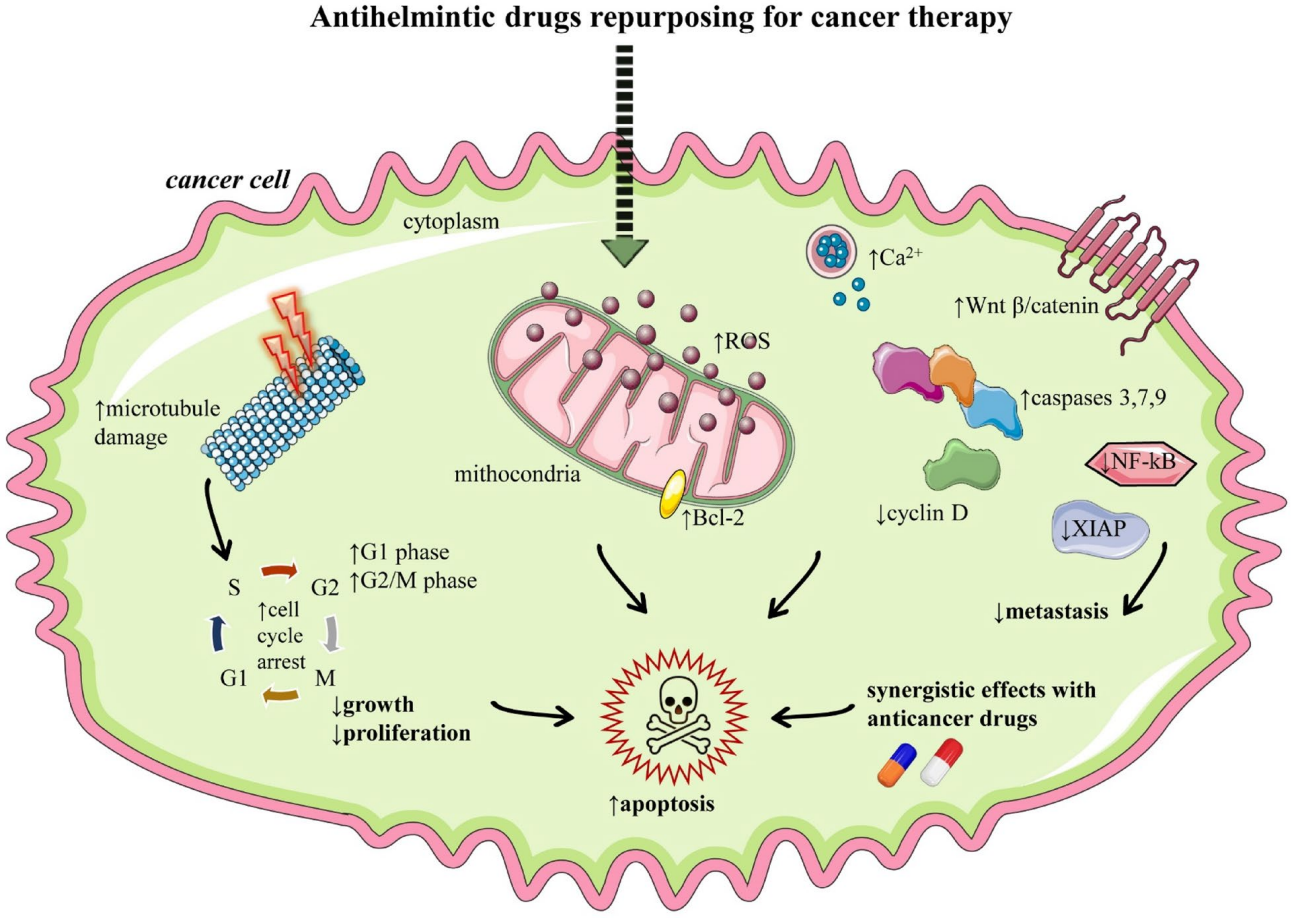
Illustrative diagram with the relevant mechanisms of repurposing anthelmintic drugs in cancer therapy. Symbols: ↑increase, ↓decrease. *Bcl-2* B-cell lymphoma 2, *ROS* reactive oxygen species, *Wnt* Wingless-related integration site, *XIAP* X-linked inhibitor of apoptosis protein

Anthelmintic are a class of drugs that are used to treat unicellular protozoa as well as parasite worms in the intestine [53]. Anti-parasitic drugs such as mebendazole, flubendazole, albendazole, ivermectin, and chloroquine are commonly used antiparasitic drugs. Initially, these drugs were used to treat cattle parasites and then subsequently they were recommended for helminthiasis in humans as well. Many studies showed that some anthelmintic drugs have beneficial effects as anticancer agents on pathways, such as activator transcription proteins, signal transducer, and nuclear factor-kappa B (NF-kB) and Wnt/β-catenin (Table 1 and Fig. 2) [53]. Therefore, these anthelmintic drugs might be potential candidates as anticancer drugs.

**Table 1:** Drug repurposing for cancer therapy

Class of drug	Name of drug	Chemical structure of drug	Type of cancer cells tested	Mechanism/results	Refs.
Anthelmintic Antiprotozoal	Flubendazole		breast cancer, leukemia, multiple myeloma, neuroblastoma colorectal cancer melanoma cells	↑Microtubule damage ↑ROS, ↑Cell cycle arrest in G2/M phase ↑Caspases 3, 7 ↑Apoptosis ↑Cytotoxicity ↓Cancer cells growth ↓Metastasis ↓Resistance to anticancer drug trastuzumab	[57–59]
	Mebendazole		breast cancer, prostate cancer colon cancer ovarian cancer thyroid cancer	Synergistic effects with docetaxel ↓Polymerization of tubulin ↑ Cell cycle arrest in G2/M phase ↑Caspase-3 ↑Apoptosis ↑Cytotoxicity ↓ Cancer cells multiplication ↓ Tumor growth ↓Metastases	[63, 65, 68, 144]
	Niclosamide		colorectal, breast, prostate and ovarian cancer	↑Cytotoxicity ↓Anaerobic metabolism, ↓glucose uptake in cancer cells ↓Signaling pathways associated with metastasis, ↓NF-κB, ↓Wnt/β-catenin, ↓STAT3	[145, 146]
	Praziquantel		colorectal adenocarcinoma, gastrointestinal cancers	↓XIAP ↓Anti-apoptotic proteins ↓Caspases ↑Apoptosis Synergic effects with paclitaxel	[53]
	Eprinomectin		prostate cancer	↑Apoptosis ↑Caspases 3, 9 ↓ROS ↑Mitotic cell arrest in G1 phase ↑Translocation of β-catenin	[87]
	Ivermectin		colon, prostate, breast and gastric cancer	↓Cancer cells growth ↓AKT–mTOR ↓Wnt/β-catenin ↓PAK1 ↓cyclin D ↓β-catenin ↓AKT/ERK//NF-kB ↓YAP1 ↓CTGF ↓EGFR	[90, 147]
	Nitazoxanide		epithelial cancer cells	↑Apoptosis ↓c-MYC ↓mTOR ↑DNA fragmentation and damage	[148]
	Clioquinol		leukemic and myeloma malignant cells	↑Apoptosis ↓HDACs ↑Cell cycle arrest ↓p53, ↓p21	[95]
	Chloroquine		pancreatic, liver cancer, cancer stem cells breast cancer	↑Autophagy, ↓Janus kinase 2 ↓DNA methylase 1 Synergistic effect in the combination with paclitaxel ↓Growth of cancer, ↓Signaling cascade of CXCL12/CXCR4	[149]
Antiviral	Ritonavir		breast, pancreatic, ovarian and lymphocytic leukemia	↓Akt phosphorylation ↑Apoptosis ↓Progression of cancer cells	[150]
	Nelfinavir		ovarian, breast, lung cancer and liposarcoma	↑Apoptosis ↓Phosphorylation of Akt ↓STAT-3 ↓Erk 1/2	[151]
	Acyclovir		breast cancer	↓Cell proliferation ↑Apoptosis ↑Caspase-3 ↓ALDH ↑Proteins expression of E-cadherin, ↓Proliferation rate, ↓Tumor growth	[125]
	Ribavirin		human lymphocytes and human squamous cell carcinoma	↓Cyclin D1 ↓Proteins cells, ↓elF4E and competing for guanylyl transferase ↓Translation of VEGF ↓mRNA, inhibit 5'-mRNA	[152]
	Cidofovir		glioblastoma and epithelial cells cancer	↑Apoptosis ↑PARP ↑Caspases ↑Cell cycle arrest in S-phase ↓DNA synthesis	[153]

Symbols: ↑increase, ↓decrease

*Abbreviations: Akt* protein kinase B, *ERK* extracellular signal-regulated kinase, *NF-Kb* Nuclear factor kappa B, *mTor* mammalian target of rapamycin, *ALDH* aldehyde dehydrogenase, *MMP-9* MATZRIX metalloproteinase-9, *Atg7* Autophagy-related E1 ligase 7, *Bax* Bcl-2-associated X protein, *Bcl2* B-cell leukemia/lymphoma 2 protein, *Bcl-XL* B-cell lymphoma-extralarge, *CAFs* cancer-associated fibroblasts, *CDC–CDK* cyclin-dependent kinase 1, *CK2* protein kinase CK2(casein kinase 2), *c-MYC* cellular myelocytomatosis oncogene, *CTGF* connective tissue growth factor, *CXCL12/CXCR4* stromal cell-derived factor-1 (CXCL12) and chemokine (C–X–C motif) receptor 4 (CXCR4) cyclin D, *DAPK* death-associated protein kinase, *DNA* deoxyribonucleic acid, *DR4/5* death receptor 4, *EGFR* epidermal growth factor receptor, *elF4E* eukaryotic translation initiation factor 4E

#### Flubendazole

Flubendazole is a well-known benzimidazole which is an antihelmintic drug that has also antineoplastic effects in different types of malignant diseases, including breast cancer, leukemia, multiple myeloma, and neuroblastoma [54, 55]. Flubendazole initiates significant changes in microtubule targeting sites, induction of apoptosis, induction of reactive oxygen species followed by G2/M phase accretion, and caspases 3, and 7 initiations in malignant cells. Flubendazole acts via different mechanisms such as inhibiting tumor growth, angiogenesis, etc. in pulmonary, liver, and breast cancer [56]. It inhibits trastuzumab resistance by targeting cancer cells, induction of apoptosis, and overexpression of human epidermal growth factor receptor 2 (HER2) in breast cancer [57]. This drug has cytotoxic activities in human colorectal cancer by blocking transcription proteins 3, signal transducer, and autophagy pathway [58]. It also blocks human melanoma cells' growth and metastasis and suppresses programmed cell death protein-1 and myeloid-derived suppressor cell accumulation [59].

#### Mebendazole

Mebendazole (MZ) is a drug that is frequently prescribed to manage gut parasitic infections. It inhibits tubulin polymerization which has an antiparasitic effect [60]. Mebendazole is a synthetic benzimidazole antihelmintic and a repositioned drug that has already proven its pharmacokinetics and toxicity profile [61]. MZ could be used in combination with temozolomide, which is a drug commonly prescribed in the treatment of malignant gliomas. In both xenograft and syngenic forms of glioma, this combination therapy suppressed tumor development more than temozolomide alone [62]. It also has synergistic effects with docetaxel inhibiting the polymerization of tubulin, mitotic arrest in the G2/M phase, augmenting apoptosis, reducing cell multiplication of prostate cancer, and suppressing tumor growth [63]. It stops mitotic growth in the G2/M phase, double-stranded breaks, and apoptosis in breast cancer which was shown using in vivo and in vitro biological assays [64]. Mebendazole activates the caspase-3 pathway and induces apoptosis. It also inhibits tumor development and stops pulmonary metastases in the later stages of thyroid cancer [65]. Mebendazole has also cytotoxic activity specific for colon cancer, ovarian cancer, endocrine malignancy, and brain tumors [62, 66, 67]. In cholangiocarcinoma, mebendazole induces apoptosis by inhibiting cell multiplication via increasing the expression of caspase-3 [68]. Other studies have found that MZ causes growth suppression in cell lines from many other different types of cancer ex-vivo and in vivo, especially pulmonary cancer [69], colorectal cancer [70], melanoma [71, 72], glioblastoma [62], and medulloblastoma [73]. The effects of MZ in melanoma are achieved by inducing apoptotic cell death, especially by activation of caspases, the pro-apoptotic Bcl-2, and suppression of the repressor of the apoptotic pathway, X-linked blocker of apoptosis (XIAP) [71, 72].

MZ has significant binding interaction potential in colon cancer cells, suggesting that it might be an antagonist of different kinases and oncogenes, such as ABL and BRAF [70], as well as a Hedgehog modulator in medulloblastoma [74]. MZ therapy in rodents caused a reduction in the size of tumors and decreased angiogenesis in comparison with normal animals. Furthermore, the incidence of metastasis in the therapy group was lower [67].

According to several case studies, MZ can also have antitumor effects in a clinical environment when given to patients with cancer [75, 76]. Mukhopadhyay et al. published one of the first studies on the antitumor effects of MZ [67]. It slowed down the growth of lung carcinoma cells but did not affect normal endothelium cells or fibroblasts. After the first- and second-line therapy in refractory tumors, MZ was given to patients with metastatic colorectal carcinoma. The patients had no adverse effects other than an increase in hepatic enzymes, and after that, the dose of the drug was reduced, and therapy efficiently eliminated practically all pulmonary and lymphovascular metastases, and partial recovery of hepatic metastases occurred as well [75]. MZ was prescribed to patients with adrenocortical cancer after the failure of different chemotherapeutics. During approximately one and a half years, there were no adverse effects, the size of the metastases decreased, and the illness was stable [76].

#### Niclosamide

Niclosamide is a drug of choice [77] and got approval from FDA as an anthelminthic drug. It has also been reported in a series of studies to have cytotoxic effects on ovarian cancer, breast cancer and prostate cancer cells [32, 78]. It targets cancer cells by interfering with the anaerobic metabolism and glucose uptake of these cells [79]. It also has an anticancer effect on multiple signaling pathways, such as metastasis, and signal activation, and as a transducer of transcription proteins Wntβ/-catenin and NF-_K_B [80, 81]. Some studies showed that it significantly suppressed the development of cancer of breast, liver, and colorectal cancer. Its anti-metastasis effect seems to prevent liver metastasis of colorectal cancer cells. It also has a beneficial effect on pulmonary metastases of breast cancer [82, 83]. However, less bioavailability and the poor solubility of the drug is the biggest obstacle to its clinical development [84]. The intravenous route might help to use the development of this drug as a repositioned drug [85, 86].

#### Praziquantel

Praziquantel is a broad-spectrum antiparasitic drug, and its mechanism of action is still unclear. Nevertheless, it can intensify the concentration of intracellular Ca^2+^ and it also causes contractions of muscles [53]. It has been shown that this drug also increases the cytotoxic action of paclitaxel at 20–40 µM. The combination of both drugs, i.e., praziquantel and paclitaxel, acts synergistically reducing the expression of anti-apoptotic protein and X-linked inhibitor of apoptosis protein (XIAP) [53].

#### Eprinomectin

Eprinomectin has a broad spectrum of effects against different parasitic infections. It showed also a cytotoxic potential against prostate cancer cells. It induces apoptosis in PC-3 cells by affecting reactive oxygen species (ROS). Eprinomectin also causes the stop of mitotic cells at the G1 phase. Moreover, this drug stimulates the translocation of β-catenin and has a significant apoptotic effect and activates caspase-3 and caspase-9. The abovementioned findings might explain why eprinomectin could have cytotoxic effects against advanced prostate cancer [87].

#### Ivermectin

Ivermectin is an FDA-approved macrocyclic lactone. It has antitumor effect in smaller doses against the Wnt-TCF, and β-catenin, and suppresses the expression of cyclin D in cancer of the colon [88]. Another approach documented the therapeutic effect of this drug in cancer xenografts and melanoma [89]. Reports on neurofibromatosis tumor cells and ovarian cancer cells suggested that this drug might suppress the growth of protein-activated kinases (PAK-1) and thus have a beneficial effect on prostate, breast, and gastric cancers [90]. This drug tends to reduce the growth of a tumor enhancing mitochondrial biogenesis compared to normal cells [91]. Similarly, in another research, it has been shown that this drug also inhibits PAK1 via AKT–mTOR in breast cancer. It reduces yes-associated protein (YAP1) expression and targets connective tissue growth factor (CTGF) in gastric cancer. It also suppresses resistance by dropping p-glycoprotein due to inhibition of ERK/AKT/NF-kB and epidermal growth factor receptor (EGFR) [92]. This drug is a self-renewal marker of cancer stem cells (CSCs) in breast cancer. Therefore, this drug could be a future therapeutic molecule for cancer management [93].

#### Nitazoxanide

Nitazoxanide is a thiazole-based molecule and it has been reported that it might have antitumor effects due to different mechanisms, such as inhibition of cellular myelocytomatosis (c-MYC), induction of apoptosis, and DNA fragmentation. It also causes autophagy against epithelial tumor cells via mechanistic targeting rapamycin (mTOR) inhibition besides its antihelmintic effects. Similarly, this drug can induce apoptosis by DNA fragmentation condensation of the nucleus along with its anti-parasitic effects [53, 94].

#### Clioquinol

Clioquinol also seems to have anticancer effects besides its antiparasitic effects. It causes downregulation expression in histone deacetylase (HDACs) in leukemic and malignant myeloma cells. This drug causes apoptosis via mitotic arrest and downregulation of HDAC, resulting in the expression of p53 and p21 [95].

#### Pyrimethamine

Pyrimethamine is an antiparasitic drug which inhibits tumor growth and metastasis in pulmonary carcinoma by attacking the dihydrofolate reductase (DHFR) and thymidine phosphorylase (TP) [96, 97]. Pyrimethamine, which is a STAT3 antagonist, has chemotherapeutic and immune-stimulatory effects in breast cancer models in mice. Pyrimethamine suppresses STAT3 action in metastatic breast cancer cell lines ex-vivo by reducing tumor growth and invasion and by increasing Lamp1 production in tumour-infiltrating CD^8+^T lymphocytes [98]. Pyrimethamine also inhibits the growth of ovarian cancer cells both ex-vivo and in-vivo [99].

The most representative mechanisms of anthelmintic drug repurposing in cancer therapy are summarized in Fig. 2**.**

### Antiviral drugs

The combinations of antiviral drugs and conventional chemotherapeutic agents are used to treat different types of malignant diseases such as lymphoma, nasopharyngeal carcinoma, hepatocellular carcinoma, and Kaposi sarcoma using protease inhibitors directed towards the human immune-deficiency virus. Studies on the viruses accompanying cancers can be a useful platform for the development of novel therapeutic approaches not only for treating viral infections but also consequently influencing tumorigeneses [100].

#### Ritonavir

Ritonavir is a thiazole-based anti-viral drug widely used for the treatment of different viral diseases such as HIV (human immunodeficiency virus) and to increase the effectiveness of protease inhibitors. Several studies have shown antitumor effects of ritonavir. It seems that it can cause apoptosis and inhibit the progression of malignant cells in breast, pancreatic and ovarian carcinoma [101–103]. The drug also strengthens the effects of several substances such as temozolomide towards glioma cancer cells [104]. It seems that it could also be used in combination with bortezomib for the treatment of renal cancer [105]. Ritonavir also has anticancer effects against breast cancer by inhibiting Akt phosphorylation and seems to be effective also in lymphocytic leukemia [106].

#### Nelfinavir

Nelfinavir is a protease inhibitor and is widely used in managing HIV-1 and HIV-2. This drug can decrease phosphorylation of Akt, signal transducer and activator of transcription 3 (STAT-3), and xenografts tumors [107] Several studies reported the beneficial effects of this drug against different types of cancers such as ovarian, breast, lungs, and liposarcoma by inhibiting the signals of Erk 1/2, STAT-3, and Akt [108–113]. It has been reported that nelfinavir induces endoplasmic reticulum (ER) stress and also increases the effect of other drugs used for the management of prostate and breast cancers [114, 115]. Numerous studies showed that it inhibits autophagy and cyclooxygenase (COX-2) inhibitors in breast cancers [116]. The mixture of COX-2 and nelfinavir inhibit autophagy and could increase cytotoxic effects directed against cancer of the breast [116]. It also induces pro-apoptotic effects by activating caspase-4 [117, 118]. Some studies found that this drug induces apoptosis mediated by ROS [119, 120]. Other studies also reported antiproliferative effects and limited toxicity against liposarcoma and cystic carcinomas [120, 121].

#### Acyclovir

Acyclovir was discovered about four decades ago and has become a drug of choice against viral infections, such as Herpes Simplex [122]. A plethora of evidence suggests that this drug is effective in the treatment of different types of cancers [123, 124]. This drug suppresses cell proliferation and induces apoptosis in malignant tumors of of the breast. Another study reported antiproliferative effects of acyclovir in Michigan Cancer Foundation (MCF-7) cell lines by increasing the proteins expression of E-cadherin, reducing the proliferation rate, and increasing apoptosis caspase-3 and wound healing in MCF-7 malignant cells. The results suggested that this drug could be used alone or in combination therapy as a potential candidate for breast cancer treatment. The results of the calorimetric assay indicated the downregulation of aldehyde dehydrogenase (ALDH) activity towards breast cancer cell lines [125].

#### Ribavirin

Ribavirin is a member of the antiviral drugs class and it seems that it has beneficial effects in different viral diseases, such as polio, hepatitis-C and SARS–coronavirus infection [126–128]. It is a guanosine ribonucleoside-based drug. Since it is similar to guanosine it tends to compete for guanylyl transferase and inhibit 5'-mRNA. This drug at micromolecular concentrations binds with eukaryotic translation initiation factor (elF4E) on a purposeful site using a 7-methyl guanosine mRNA cap. This drug suppresses elF4E facilitated oncogenes transformation using 7-methyl guanosine. In another approach, it has been found that it can inhibit elF4E competitive binding to cyclin D1 and thus reduce cyclin D1 protein cells [124]. This drug also causes the translation of VEGF mRNA and other genes [129, 130]. Ribavirin at 2 µm concentration suppresses the progression of human lymphocytes [131].

#### Cidofovir

Cidofovir is a FDA accepted nucleoside broad-spectrum antiviral drug which is used to treat different viral infections by inhibiting the viral DNA polymerase through its metabolite diphosphate [132–134]. Because of this mechanism, the drug can decrease the growth of several types of human cancers [135–139]. This drug also induces apoptosis mitotic arrest in S-phase and causes activation of Poly (ADP-ribose) polymerase (PARP) and caspase in epithelial cells [140–142]. It also inhibits DNA synthesis and blocks the growth of cancer cells. It has also been reported that this drug has cytotoxic effects on human glioblastoma cell lines. This drug shows antiproliferative effects both in xenograft models and in vitro studies. It inhibits the gene expression associated with apoptosis in glioblastoma. This drug has antitumor effects because of its different mechanisms, including effects on mitogenic pathways and activation of proapoptotic pathways in glioblastoma [143].

### Antibiotics

#### Sulfisoxazole

Sulfisoxazole is an antibiotic that may inhibit small extracellular vesicular exudation from cancerous mammary cells by interfering with endothelin receptor A. In animal studies of breast cancer xenografts, sulfisoxazole had antitumor and antimetastatic effects (Table 2) [154].

**Table 2:** Antibiotics, antifungals, antimalarial, anti-inflammatory as potential drug candidate against cancer

Class of Drug	Name of drug	Structure	Type of cancer to be used	Mechanism	Refs.
Antibiotics	Sulfisoxazole		Breast cancer	Interfering with endothelin receptor A to stop breast cancer cells from exuding tiny extracellular vesicles	[154]
	Azithromycin		Colon cancer	↑TNF-α-related apoptosis ↑TRAIL ↑DR4/5 ↓Autophagy	[155]
	Doxycycline		Osteosarcoma, prostate carcinoma, myeloid and colon cancer	In myeloid and colon cancer, it prevents permeation by lowering MMP-2 and MMP-9	[166]
	Anthracycline		Endometrial, breast, bladder, hepatic, thyroid, and pulmonary malignancies	Attaches to DNA, causing it to get alkylated, which stops the cell cycle	[182]
Antifungals	Itraconazole		Non-small cell lung cancer	By eliminating lipids from the plasma membrane, it decreases AKT1 activity, which inhibits its downstream target mTOR, resulting in mortality and growth slowering	[188]
	Rapamycin		Breast cancer	It promotes intracellular autophagy and boosts the function of Atg7 and DAPK via transcriptional activation	[196]
	Griseofulvin		Colorectal and cervical cancers	Affects microtubule assembly in MCF-7 cells, causing programmed cell death ↑Cell cycle arrest	[199–201]
	Clotrimazole		Breast, colon and pulmonary cancer	Blockes actin polymerization and activates glycolytic inflow	[203]
	Ciclopirox		Breast, colorectal cancer rhabdomyosarcoma	↓CDC–CDK, ↓Bcl-XL ↑Caspase-dependent cascade causing apoptotic cell death	[208, 209]
	Nannocystin A		Colorectal and breast cancer cells	Target eukaryotic elongation factor 1 in proteome investigations	[213, 214]
NSAIDS drugs	Aspirin		Hepatocellular carcinoma	Affects P4HA2 by suppressing NF-κB and LMCD1-AS1/let-7g of Aspirin prevents tumour growth and accumulation of collagen	[313, 314]
	Ibuprofen		Adenocarcinoma	Modulates the expression levels of cancer-related genes Akt, P53, PCNA, Bax, and Bcl2	[228]
	Naproxen		bladder carcinoma	↑Cell arrest ↑Cancer cells death ↑PI3K	[233]
	Diclofenac		Ovarian cancer	↑Apoptotic cell death ↓SOD2 ↓Proportion of free radicals	[243, 244]
	Celecoxib		Bladder cancer	Blocks epithelial-to-mesenchymal transformation ↓miRNA-145/TGFBR2/Smad3 axis	[250]
	Indomethacin		Colon cancer	↓Cancer cell proliferation ↓PKC-p38-DRP1 ↓Wnt/-βcatenin signalling, to effectively target MAPK mechanisms	[259, 263]
	Thiocolchicoside		Leukaemia, lymphoma, and squamous cell carcinoma	Blocks the receptor stimulator NF-kB ligand ↓NF-kB signalling cascade ↓Cancer-induced bone metastasis	[308, 309]
	Artemisinin		Breast cancer	Deactivates cancer-related fibroblasts and decreases CAFs mediating growth and metastases by suppressing TGF-β signalling	[315]
	Artesunate		Hepatocellular carcinoma	↑Pro-apoptotic proteins ↑caspases ↓MYC oncogene ↓Anti-apoptotic proteins	[272, 273]
	Dihydroartemisinin		Ovarian cancer	↓Cancer cell development ↓Metastases by addressing the platelet-derived growth factor receptor-alpha (PDGFR)	[316]
	Mebendazole		Melanoma	↑Apoptotic cell death ↑Caspases ↑Bcl-2 ↓Repressor of apoptosis X-linked blocker of apoptosis (XIAP)	[71, 72]
	Chloroquine		Metastatic tumors	Par-4-dependent suppression, mediates p53- and Rab8b-based Par-4 production to promote tumour cell death	[279]
	Pyrimethamine		Non-small cell lung cancer	↓EMT ↓Invasion ↓Cancer cells growth, ↓Metastases by interacting with dihydrofolate reductase and thymidine phosphorylase	[96, 97]
	Quinacrine		Renal cancer	Facilitates chromatin transcription (FACT) protein complex, which seems to be trapped on chromatin and induces CK2-induced phosphorylation of p53, responsible for quinacrine-mediated p53 transcription	[299, 300]

Symbols: symbols: ↑increase, ↓decrease

*EMT* epithelial-to-mesenchymal transition, *Erk ½* extracellular signal-regulated kinase 1/2, *HDACs* histone deacetylase inhibitors, *M Phase* mitosis, *m RNA* messenger ribonucleic acid, *MAPK* mitogen-activated protein kinase, *MCF-7* michigan cancer foundation-7, *miRNA* microRNAs, *MMP-2* matrix metalloproteinases-2, *MYC* master regulator of cell cycle entry and proliferative metabolism, *NF-kB* nuclear factor kappa-light-chain-enhancer of activated B cells, *P4HA2* collagen prolyl-4-hydroxylase α subunit 2, *PARP* poly ADP ribose polymerase, *PCNA* proliferating cell nuclear antigen, *PDGFR* platelet-derived growth factors, *PI3K* phosphoinositide 3-kinases, *ROS* reactive oxygen species, *SOD2* superoxide dismutase 2, *STAT-3* signal transducer and activator of transcription 3, *TGFBR2* transforming growth factor-beta, *TRAIL* TNF-related apoptosis-inducing ligand, *VEGF* vascular endothelial growth factor, *XIAP* X-linked inhibitor of apoptosis protein, *YAP1* yes-associated protein 1

#### Azithromycin

Azithromycin is a macrolide antibacterial drug. The proliferative potential of cancer cells has been inhibited by this drug. In colon carcinoma cells, ex-vivo and in-vivo, it increases the antineoplastic effectiveness of TNF-α-related apoptosis-inducing ligand (TRAIL) by suppressing autophagy and increasing DR4/5 [155]. Azithromycin also reduces angiogenesis in pulmonary carcinoma by inhibiting vascular endothelial growth factor receptor 2-induced focal adhesion and the PI3K/AKT signalling cascade [156].

#### Doxycycline

Doxycycline is a tetracycline antibiotic that is used for treating different infections. Some tetracyclines were reported to suppress angiogenesis in the early 1990s [157], and doxycycline was eventually found to have antiproliferative activity in bone and prostate carcinoma and mesothelioma cells [158–160]. It has also been shown to induce apoptosis in pancreatic islets [161, 162] and myeloid cells [163]. Matrix metalloproteinases (MMPs) are inhibited by tetracyclines [164]. Doxycycline therapy inhibits penetration by downregulating MMP-2 and MMP-9 levels in myeloid cells [165] and colon carcinoma [166]. Doxycycline has been investigated as a suppressor of tumour progression, because MMPs are assumed to play a crucial role in cancer infiltration and progression. Doxycycline has been shown to reduce different tumours and it seems to be beneficial for breast cancer patients at risk for osteolytic bone metastasis. [167]. The same investigators also demonstrated that zoledronic acid, a medicine used to decrease the incidence of bone fractures in patients with osteosarcoma or osteoporosis, might be useful in combination with doxycycline [168]. MMP-2/9 suppression has also been shown to reduce metastasis in preclinical trials of prostate cancer [169] and squamous skin carcinoma [170]. Doxycycline can reduce EMT-marker transcription in pulmonary and hepatic carcinoma cells, reversing their pro-metastatic character [171, 172]. Doxycycline therapy reduced clonogenic potential and decreased the expression level of stem cell markers in hepatic carcinoma cells enriched with stem-related characteristics. Doxycycline decreased proliferation markers Ki67 and PCNA in the in vivo xenograft mouse model. [173]. Doxycycline was combined with interferon-alpha (IF-α) treatment in a phase II clinical study of renal cancer metastases. VEGF levels were measured to see if there was an antiangiogenic effect. The combined therapy proved to be ineffective in patients with renal carcinoma metastases, despite modest early reduction of VEGF expression in some patients [174]. The efficacy of doxycycline therapy in combination with bone-targeting medicines was evaluated in a recent phase II study in females with breast carcinoma metastases. In this trial, doxycycline was shown to have a negligible effect but it was associated with severe harmful effects [175].

#### Ionophore antibiotics

Ionophore antibiotics have shown antitumor effects against cancers of the colon, and prostate, as well as endometrial, blood, cerebral, and bone malignancies. Ionophore antibiotics salinomycin and nigericin specifically attack CSCs and it seem that they are slightly more effective than paclitaxel. Cell migration, metastases, and the GTPase K-Ras cascade are all targeted by these drugs. Salinomycin inhibited the hedgehog and WNT/-β catenin pathways, resulting in decreased tumour size of metastatic breast cancer [176]. In lymphomas, salinomycin combined with doxorubicin was reported to have a synergistic effect [176]. Rapamycin, an antimicrobial drug, has an antiproliferative effect by blocking cell cycle progression due to its effect on CDK proteins and mTOR signalling. In vivo investigations have shown that it can also prevent cancer and malignancies caused by the Epstein–Barr virus [177]. Rapamycin has chemotherapeutic potential in different types of human cancers, and it was found to synergize with erlotinib in NSCLC and paediatric glioma [177, 178].

#### Anthracycline drugs

Anthracycline drugs have also been intensively studied as antimalignant drugs. Doxorubicin, idarubicin, mitoxantrone, daunorubicin and epirubicin are often used anthracycline medicines that have been extensively investigated in solid and blood malignancies [179–181]. Garg et al*.* found that doxorubicin and selinexor promoted the death of thyroid cancer and AML cells when used together [180, 181]. Duocarmycin is an antibiotic that works at low doses. It attaches to the DNA molecule causing DNA to be alkylated, which causes the cell cycle arrest. It is one of the most successful medications used to treat endometrial, breast, bladder, hepatic, thyroid, and pulmonary malignancies [182]. Wang et al. analyzed 124 patients from January 1996 to July 2018 to analyze the effects of chemotherapeutic treatment with gemcitabine and anthracycline (epirubicin and pirarubicin). They analyzed the probability of tumour’s recurrence and therapy failures. Gemcitabine had a lower recurrence percentage and therapy failure rates than anthracycline antimicrobials, suggesting that this approach should be explored for patients who cannot be treated with BCG [183]. Landomycin E is an angucycline antibiotic produced by Streptomyces globisporus which caused cell death in T-cell leukaemia cells by rapidly generating hydrogen peroxide and activating caspases [184]. NAC, in combination with doxorubicin, demonstrated decreased adverse effects on nephrons, somewhat enhanced cytotoxic effects of T-cells, and it slightly enhanced their survivability. NAC, in combination with landomycin, on the other hand, significantly enhanced the lifespan of the rodents while also having some tissue-protective effects. As a result, NAC in combination with landomycin appears to be more effective than doxorubicin. Landomycin seems to be more powerful than doxorubicin at low concentrations in ex-vivo and in-vivo melanoma with fewer adverse effects. However, adverse effects such as cardiovascular events and mucositis suggested the need for other antibacterial drugs. Idarubicin, a doxorubicin analogue, was created. In acute myelogenous leukaemia, idarubicin showed increased lipophilicity and anti-malignant efficacy. In acute myeloid leukaemia clarubicin, an anthracycline antibiotic derived from *Streptomyces galilaeus*, suppresses RNA production. Amrubicin has chemotherapeutic efficacy against SCLC, lymphoma cells and bladder carcinoma and got marketing authorization in Japan. Zorubicin which is a benzoylhydrazone derivative of the well-known anthracycline antineoplastic antibiotic daunorubicin is at the moment in the confirmatory phase of clinical studies for breast cancer and leukaemia.

### Antifungals

#### *Itraconazol*e

**Fig. 3:**
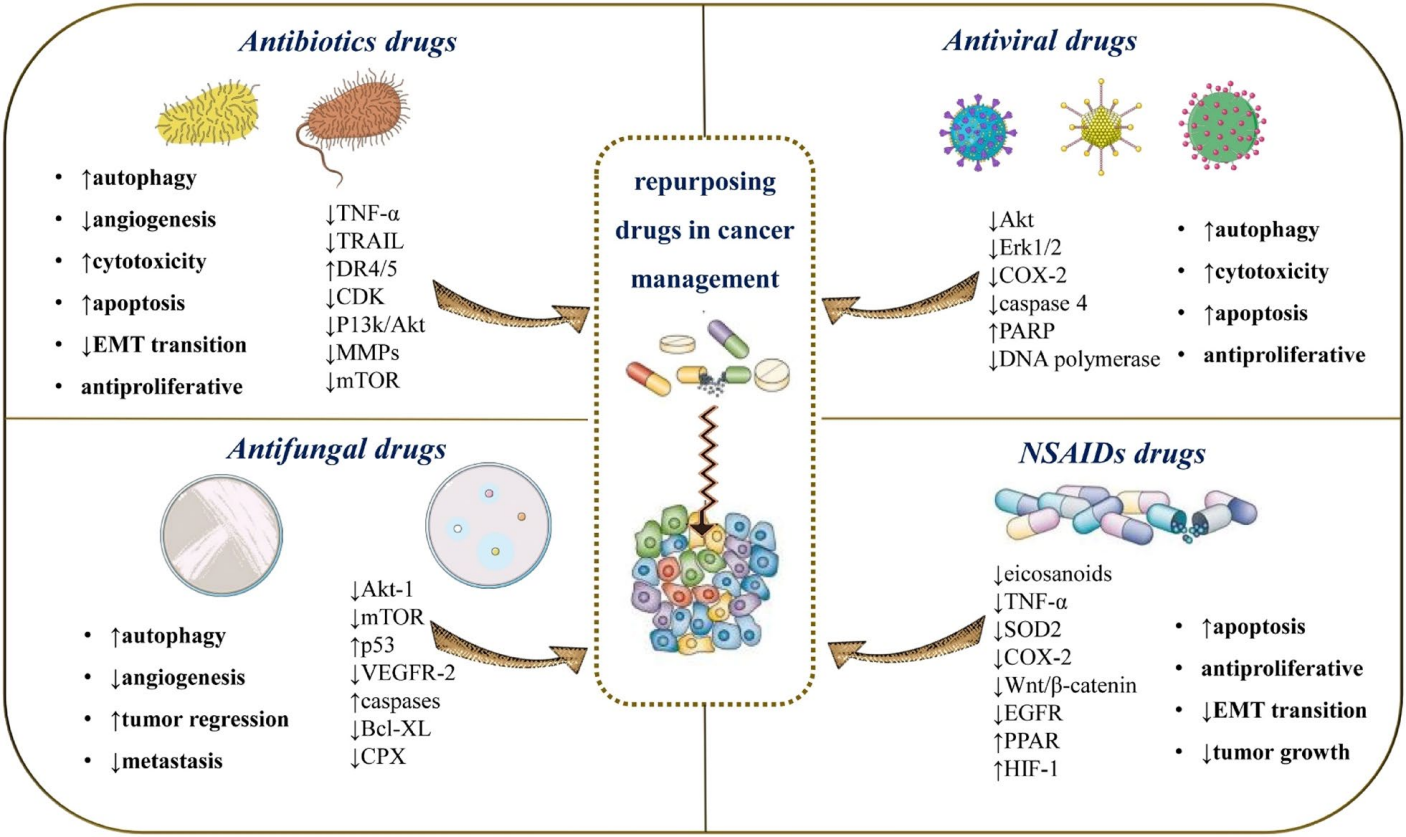
Schematic representation of the most representative drug clases used as repurposing drugs in oncology. Symbols: ↑increase, ↓decrease. *Akt* protein kinase B, *Bcl-2* B-cell lymphoma 2, *Bcl-xL* B-cell lymphoma-extra-large, *CDK* cyclin-dependent kinases, *COX-2* Cyclooxygenase-2, *CPX* cyclopirox olamine, *DR4/5* death receptor 4/5, *EGFR* epidermal growth factor receptor, *EMT* epithelial–mesenchymal transition, *Erk1/2* extracellular signal-regulated kinase 1/2, *HIF-1* hypoxia-inducible factor-1, *MMPs* matrix metalloproteinase, *mTOR* mammalian target of rapamycin, *NF-KB* nuclear factor kappa B, *P53* TP53 or tumor protein 53, *PARP* Poly (ADP-ribose) polymerase, *PI3k/Akt* phosphoinositide-3-kinase–protein kinase B/Akt, *PPAR* peroxisome proliferator-activated receptor, *ROS* reactive oxygen species, *SOD2* superoxide dismutase, *TNF-α* tumor necrosis factor alpha, *TRAIL* TNF-related apoptosis inducing ligand, *VEGFR-2* vascular endothelial growth factor receptor-2, *Wnt* wingless-related integration site, *XIAP* X-linked inhibitor of apoptosis protein

Itraconazole is an antifungal drug that works by inhibiting 14-α-lanosterol demethylase (14-LDM), a crucial enzyme in cholesterol production. Itraconazole also decreases angiogenesis in endothelial cells by reducing endothelial growth [185] and suppresses VEGFR-2 levels and multiple cellular pathways in endothelial cells [186]. Treatment with itraconazole of human vascular endothelial cells (HUVECs) inhibited movement and tube creation by decreasing the phosphorylation of growth factor receptors [187]. Itraconazole therapy of pulmonary carcinoma xenografts reduced angiogenesis and regression in this study. Its antitumor activity may be mediated by different pathways together with its antiangiogenic effects. In glioblastoma cells, removing lipids from the plasma membrane reduced Akt-1 action, which inhibited its downstream target mTOR, causing death and decreased tumor growth [188] (Fig. 3). It has been hypothesized that itraconazole might be a Hedgehog antagonist. In mice treated with itraconazole the proliferation of two hedgehog-based tumor types, a medulloblastoma and a basal cell cancer of the skin, was reduced in vivo [189]. Research on pleural mesothelioma cells came up with similar results [190]. Itraconazole was to a certain extent successful in a randomized clinical study with metastatic castration-resistant prostate carcinoma, with an extended PSA progression-free life, and suppression of Hedgehog signalling [191]. Itraconazole was also used to treat 19 patients with basal cell malignancy in a limited phase II study. Hedgehog signalling was decreased which was followed by slower tumor growth and tumor regression. In a minor phase II trial, combining itraconazole with conventional anticancer therapy for lung cancers improved both progression-free and ultimate survival. The authors of this study speculated that this result could be attributed to the antiangiogenic effects of itraconazole [192]. Nevertheless, there might be some problems with itraconazole treatment of malignant diseases. According to some studies, antifungal medications may affect the effects of other anticancer drugs, particularly antibodies, such as rituximab [193].

#### Rapamycin

Rapamycin, also called “sirolimus,” is a drug that was first discovered for its potent antifungal effects [194]. Because of its unique immunosuppressive effects, rapamycin has been routinely used to prevent rejection after organ transplantation. Rapamycin has relatively recently been identified as an mTOR inhibitor that may be used to treat Kras Pten endocrine ductal adenocarcinoma, resulting in inhibition of proliferation and tumor size shrinkage [195]. By transcriptional activation of Atg7 and DAPK, rapamycin was also used to activate cellular autophagy and increase the chemotherapeutic effects of dihydro-artemisinin in breast tumor cells [196]. Due to the increased expression of p73, rapamycin improves the susceptibility of ER-positive breast cancer cells to tamoxifen [197].

#### Griseofulvin

Griseofulvin causes apoptotic cell death in lymphoma and leukemia cells. [198]. It affects microtubule assembly in MCF-7 cells causing apoptotic death and cell cycle arrest, and it has a stimulatory effect together with vinblastine [199]. The same chemotherapeutic effects have been observed in colorectal and cervical cancers [200, 201]. Centrosome clumping has also been associated with the production of micronuclei in prostate carcinoma. In pulmonary and prostate cancer, combining radiation with griseofulvin therapy showed synergistic anticancer effects. The sulfonyl group substitution analogues of griseofulvin have antiproliferative effects on oral cancer cells and cytotoxic activity on breast cancer cells [202].

#### Clotrimazole

Clotrimazole inhibits glioblastoma cell invasion and metastasis. It inhibited actin polymerization and promoted glycolytic inflow in breast, colon, and pulmonary cancer cells [203]. Kadavakollu et al. [204] evaluated many potential anti-cancer pathways. In prostate and cervical cancers, as well as lymphoid malignancies, ruthenium combined with other drugs, had stronger cytotoxic activity than the monotherapy with individual drugs [205]. A combination of imatinib and clotrimazole inhibited the glycolysis pathway more effectively and boosted NO and VEGF production in breast cancer cells [206].

#### Ciclopirox

Ciclopirox (CPX), an antifungal drug, induced ageing in p53 deficient HeLa cells by a mechanism unrelated to mTOR [207]. Due to degrading CDC–CDK, downregulating Bcl-xL, and activating the caspase-dependent cascade, it causes apoptotic cell death in breast and colorectal cancer, and rhabdomyosarcoma cells [208, 209]. Prolonged exposure to this drug caused p53-independent caspase stimulation and cell death [207]. It has also been demonstrated that it suppresses HPV genetic mutations. By activating oxygen radicals, caspase-3, and lowering Bcl-xL levels, CPX was significantly more effective than gemcitabine in endocrine carcinoma. Nevertheless, when it came to triggering apoptotic pathways, the combination of these drugs was significantly more efficacious than each of these medicines alone [210]. According to ex-vivo and in-vivo studies in colorectal cancer, CPX causes autophagy through the depletion of DJ-1 and the formation of oxygen radicals [211]. According to Ahmad et al. ethacrynic acid and CPX had an additive anticancer effect in hepatocellular carcinoma. However, low concentrations of CPX were harmful not just to cancer cells but also to healthy cells [212].

#### Nannocystin A

Nannocystin A showed chemotherapeutic effects on colorectal and breast cancer cells. Nannocystin A has been shown to target eukaryotic elongation factor 1 in proteome studies [213, 214]. These findings support the repositioning of an antifungal drug with anticancer potential. In future, additional clinical testing is needed to confirm this.

### NSAIDS/anti-inflammatory drugs

#### Aspirin

Aspirin is a drug that has effects on cyclooxygenase (COX) isoenzymes 1 and 2 and is widely used in the treatment and prevention of myocardial infarction in patients with coronary heart disease. COX-1 is important for platelet synthesis of thromboxane A2, which results in platelet aggregation and adhesion to cells, including malignant cells. Platelets covering tumor cells prevent the immune system from recognizing these cells, favoring the development and spread of cancer. COX-2 is important in the production of prostaglandin E2, which significantly stimulates the growth of tumor cells [215–217]. Recent pharmacological studies have analyzed the potential of aspirin as a therapeutic approach in spontaneous or chemically provoked tumors [218–220]. Many clinical trials have found that taking aspirin after a diagnosis is associated with a better prognosis in patients with colorectal cancer [221–224]. However, because most of these studies were retrospective and the patient recruitment was not homogenous, there is a lot of contradictory information. To determine the function of aspirin as a potential anticancer therapy, prospective trials are required. Several clinical trials are now in progress, the majority of which are looking at the effect of aspirin in preventing relaps of the illness.

#### Ibuprofen

Ibuprofen is a non-selective cyclooxygenase inhibitor. It slows down the development of prostate carcinoma [225]. It also has a radio-sensitizing effect ex-vivo but at larger doses than those that have been documented to suppress eicosanoid production, indicating that other pathways are included [226, 227]. Anti-angiogenesis, initiation of apoptosis, and decrease of cellular proliferation were found to have antitumor effects on gastric adenocarcinoma cells ex-vivo, along with modulating the expression levels of the cancer-related genes Akt, PCNA, Bax, P53, and Bcl2 [228] (Fig. 3). It has been shown that TNF-α upregulated metastatic melanoma cell migration in vitro and that this could be reduced by ibuprofen both in solution and delivered from a hydrogel. Although this might be attributed to the induction of apoptotic cell death, the mechanism of this is still not completely explained [229, 230]. Chemosensitivity could also be modulated by ibuprofen. Ibuprofen therapy reduced the amounts of Hsp70, a heat shock protein associated with apoptotic tolerance in lung carcinoma cells. Following ibuprofen therapy, blocking Hsp70 and inducing apoptosis improved responsiveness to the anticancer drug, cisplatin [231].

#### Naproxen

Naproxen is a propionic-acid analogue that reduces cell growth, provokes programmed cell death, and restricts metastasis [232]. This drug is a non-selective cyclooxygenase inhibitor. Ex-vivo and in-vivo chemotherapeutic effects have been documented in breast, leukemic, bladder, colorectal, and osteosarcoma cells. Naproxen causes the death of bladder carcinoma cells ex-vivo by targeting PI3K [233, 234]. A combination of cholesterol-lowering drug atorvastatin and naproxen effectively suppressed colon adenocarcinomas in experimental animals in-vivo [235]. A combination of calcitriol and naproxen has been tested in phase II clinical trials for preventing the relapse of prostate carcinoma. Such a combined therapy has been well-tolerated, with 19% of enrolled participants having a reduction in PSA doubling time (PSADT) and 67% having a prolongation of PSADT when compared with baseline [236].

#### Diclofenac

Diclofenac is an acetic acid derivative with a modest affinity for cyclooxygenase-2. It has been shown that this drug has beneficial effects on several malignant tumors including fibrosarcoma, hepatoma, colorectal, endometrial, and endocrine carcinoma. The effect of diclofenac (3%) and calcitriol was shown on different carcinoma cancer cell types, such as endometrial, breast, cerebral, colorectal, endocrine, non-small cell lung cancer, hepatic, and showed a higher cytotoxic effect in cells from chronic lymphocytic leukemia than in normal lymphocytes[237, 238]. Furthermore, growth rates and degree of vasculature were significantly decreased in experiments on rats with fibrosarcoma and hepatoma [239]. Diclofenac also has an anticancer effect in colon carcinoma [240, 241]. Diclofenac also reduced tumor growth in a mouse model of pancreatic cancer [242], as well as in ovarian cancer [243]. In vitro evidence suggests that diclofenac therapy induces apoptotic cell death by inhibiting antioxidant SOD2, resulting in a greater proportion of free radicals [244]. Despite the increasing amount of data supporting the anticancer effects of diclofenac, there are at the moment no active clinical studies evaluating the effects of diclofenac as a chemotherapeutic agent. A phase II clinical study for basal cell carcinoma, on the other hand, was just completed. Diclofenac was tested as a stand-alone treatment and in combination with calcitriol. The study found that diclofenac applied topically was more successful than combination therapy, with complete histologic tumor regression in 64.3% [245].

#### Celecoxib

Celecoxib, a specific COX-2 blocker, has chemotherapeutic effects in different types of cancer. In randomized controlled studies celecoxib combined with chemotherapeutic treatment has beneficial effects on breast cancer, progressive pulmonary carcinoma, and transitory bladder cancer [246–249]. Through miRNA-145/TGFBR2/Smad3 axis, celecoxib suppresses the epithelial-to-mesenchymal shift in cancer bladder cells [250]. Celecoxib decreases liver cancer cells proliferation and metastasis by addressing PNO1 and reduces AKT/c-Met-induced hepatocarcinogenesis by inhibiting COX-2/Akt/FASN pathway [249, 251, 252]. Celecoxib has effects on proline metabolism, generating an upregulation in proapoptotic markers (PRODH/POX, PPAR), lowering HIF-1 levels, and triggering squamous skin carcinoma programmed cell death. In human oral cancer cells, combination therapy with celecoxib and calyculin-A suppresses epithelial–mesenchymal shift [253, 254].

#### Indomethacin

Indomethacin is an antinociceptive non-steroidal anti-inflammatory drug (NSAID) widely used in the management of rheumatoid diseases [255]. It has been noted that patients who were treated for a long period with NSAIDs had a decreased chance of acquiring cancer which was confirmed in several clinical studies [255]. Furthermore, there is a growing number of publications suggesting that indomethacin and indomethacin-dependent prodrugs have chemo-preventive effects against different malignant diseases by inhibiting COX-1/2-associated angiogenesis [256, 257]. Indomethacin inhibits cancer cell progression by competing with calcium-related signalling and the creation of focal interactions [258]. A COX-independent mode of activity for indomethacin's antiproliferative effect has been discovered in several studies, as indicated by cell growth suppression in indomethacin-treated colonic cancer cells that would not exhibit COX-1/2. [259–261]. Lin et al. relatively recently proposed that the chemotherapeutic activity of indomethacin might be due to MAPK-associated pathway suppression [262]. They used computational scanning to perform this drug repurposing using a current drug repository. Indomethacin was proven to have a stronger association with ShcPTB by interacting with the phosphotyrosine binding (PTB) region of adaptor protein Shc (ShcPTB) as a readout. It seems that indomethacin competes against active EGFR by interacting with ShcPTB without disrupting the ERK-binding region, preventing EGFR from recruiting Shc and inducing abnormal signalling as a consequence of ERK production. Indomethacin suppresses cancer cell proliferation by disturbing PKC–p38–DRP1 axis-based mitochondrial dynamics or downregulating Wnt/β-catenin signalling to effectively target MAPK mechanisms [263]. NSAIDs, such as indomethacin, are currently seriously being considered as potential anti-carcinogenic medicines [264–267]. The therapeutic use of indomethacin offers a lot of potentials particularly if data would be collected enabling a better knowledge of the processes concerning its antiproliferative effects.

### Antimalarial drugs

#### Artemisinins

A drug development initiative for the management of malaria led to the innovation of artemisinins. Artemisinins are plant extracts that have been used in Chinese traditional medicine for centuries. 

When used as a treatment for malaria, artemisinins trigger the production of free radicals in infected erythrocytes, eradicating the plasmodium parasite [268]. The effects of artemisinins are attributed to the interaction of endoperoxide moiety with the Fe-containing heme groups in the affected RBCs [269]. Artesunate is by far the most researched artemisinin which could be used for cancer medication repurposing. Ex-vivo and in-vivo data indicate that it might have antiangiogenic effects, the effects on the formation of free radicals, and the modification of antimicrobial resistance in a wide range of cancers [270]. Artesunate has antiproliferative and pro-apoptotic effects on lymphoma and myeloma cells [271], as well as on hepatocellular cancer cells [272, 273]. An increase in the expression of pro-apoptotic proteins, such as caspase-3, a reduction in the MYC oncogene, and a reduction of many anti-apoptotic proteins are all possible explanations for why this drug might have anti-malignant effects. The chemotherapeutic efficacy of artesunate in hepatocellular cancer is increased when combined with sorafenib [272] and gemcitabine [273]. Antiangiogenic effects of artesunate have been documented in renal cancer and hepatocellular carcinoma, with decreased tumor development in vivo, lower vessel number, and decreased vascular endothelial growth factor [274]. 

Dihydroartemisinin (DHA) is an analogue of artemisinin that inhibits leukemia cell proliferation by inducing autophagy and programmed cell death which is reactive oxygen species-dependent [275]. Decreased expression of the protein transferrin receptor 1 (TfR1) and cell cycle arrest were two factors that contributed to the effect of DHA. DHA therapy showed a significant reduction in iron in hepatoma and breast cancer cells due to the downregulation of TfR1, a protein that plays a significant role in iron absorption [276]. In leukemic cells, the TfR1 level was reduced [277]. TfR1 level was required for DHA responsiveness in different papillomavirus-infected cells and cervical cancer cells. In an in-vivo papilloma model, DHA therapy also suppressed tumour development [278].

#### Chloroquine

Chloroquine (CQ) is an antimalarial drug which was developed in the 1930s became the most widely used synthetic antimalarial drug during the 1960s and 1970s until the development of newer antimalarials in the whole world. Chloroquine has relatively recently been found to have potential anticancer effects. Chloroquine triggers tumor apoptosis by p53 and Rab8b-dependent Par-4 release, resulting in Par-4-dependent suppression of metastatic tumor development [279]. By reverting tumour-based macrophages to the M1 phenotype, chloroquine has effects on anticancer immune system response [280]. Chloroquine inhibits extrinsic neutrophil entrapment which decreases hypercoagulability in pancreatic malignant tumors [281]. Chloroquine suppresses tumour-associated Kv10.1 gates and reduces MDAMB-231 breast carcinoma cell motility ex-vivo [282]. In colon cancer, the combination of temsirolimus and chloroquine improves radiosensitivity [283]. Chloroquine sensitizes human T-cell blood cancer with oncogenic NOTCH1 mutations to secretase reduction [284]. Chloroquine is a derivative of quinoline similar to that of clioquinol. Many studies have reported its antitumor effects both in-vivo and in vitro. It has a synergistic effect in combination with paclitaxel and can stop the growth of breast malignant tumors [285]. Chloroquine, in combination with gemcitabine, seems to be able to eradicate tumor cells in xenograft models [149].

#### Hydroxychloroquine

Hydroxychloroquine is an analogue of chloroquine which has the same therapeutic effects as chloroquine although with less systemic toxicity. Hydroxychloroquine blocks intracellular lysosomal activities and improves the anticancer activities of breast cancer and glioblastoma [286, 287]. Hydroxychloroquine is an autophagy blocker that significantly increases the chemotherapeutic activity of bevacizumab on glioblastoma by suppressing of autophagy [288]. Ex-vivo and in-vivo, hydroxychloroquine increase the chemotherapeutic potential of the anti-angiogenesis drug BC001, suppressing the development of gastric carcinoma [289]. Pulmonary cancer cells are suppressed by hydroxychloroquine due to the increased chemo-sensitizing effect and effects on the change of M2-TAMs to M1-related macrophages, which improves the CD^8+^ T cell immunological reaction [290].

#### Quinacrine

Quinacrine was first identified in the 1920s as an antimalarial drug [291]. It is also used as an antibiotic, and a pleural sclerosing substance to manage giardiasis—a protozoal infection of the intestinal tract, certain types of lupus erythematosus and rheumatoid arthritis [292–294]. Quinacrine has also been used in clinical studies for the management of Creutzfeldt–Jakob disease including a new variant of CJD which is linked to contamination of food by the bovine spongiform encephalopathy (BSE). Quinacrine seems to be very suitable for repurposing for cancer therapy [295–297]. Earlier studies have shown that quinacrine has beneficial effects on different malignancies and that these effects are mediated by p53 activation. Quinacrine promotes p53 expression in renal cancer using suggesting that simultaneous inhibition of NF-kappaB and activation of p53 by a single small molecule can have anti-cancer effects [298]. It seems that quinacrine's cytotoxicity is associated with elevated p53 levels. It also seems that the facilitates chromatin transcription (FACT) protein complex, which seems to be trapped on chromatin and induces CK2-induced phosphorylation of p53, is responsible for quinacrine-mediated p53 transcription [299, 300]. Conversely, there are indications that the down-regulation of p53 increases the quinacrine effect in MCF-7 cells when compared with normal cells [301]. Nonetheless, the results of some studies suggest that quinacrine cytotoxicity on cancer cells is influenced by the p53 expression, at least to a certain extent [302, 303]. Studies with quinacrine as a chemotherapeutic drug are published recently quite often [32]. For example, in a Phase, I and Phase II clinical study, Fox Chase Cancer Center researchers combined quinacrine with capecitabine to treat colon carcinoma (NCT01844076). In the Phase I clinical study, quinacrine was combined with erlotinib for the management of recurrent or delayed pulmonary cancer (NCT01839955). Overall, quinacrine is very promising as an anticancer treatment, and the effects of this drug might be associated with the stimulation of p53, a crucial growth inhibitor that is dysregulated in many malignant diseases [32].

#### Atovaquone

In recent years, Atovaquone, a well-studied molecule known for its role as a non-oncological and anti-malarial drug, has garnered significant attention in the field of cancer therapy [304]. With its established safety profile and extensive clinical use, Atovaquone has emerged as a potential candidate for repurposing in the treatment of cancer [305]. Atovaquone, primarily used as an antiparasitic agent against malaria, has demonstrated promising anticancer properties in preclinical studies. Multiple investigations have shown its ability to inhibit cancer cell growth and induce apoptosis in various cancer types, including lung, breast, colon, and prostate cancers. These findings highlight the potential of Atovaquone as an effective anticancer agent [305].

Mechanistically, Atovaquone exerts its anticancer effects through multiple pathways. It has been shown to disrupt mitochondrial function, leading to energy depletion and apoptosis in cancer cells [304]. In addition, Atovaquone has demonstrated the ability to inhibit specific signaling pathways involved in cancer cell proliferation and survival, such as the PI3K/AKT pathway [306]. Moreover, the favorable safety profile of Atovaquone, established through its extensive use as an anti-malarial agent, further supports its potential for repurposing in cancer therapy. The well-tolerated nature of Atovaquone could potentially minimize adverse effects commonly associated with traditional chemotherapy agents, offering a more favorable treatment option for cancer patients [304, 306]. Although the repurposing of Atovaquone for cancer therapy is still in its early stages, ongoing preclinical and clinical studies are actively investigating its efficacy and safety in cancer treatment [304]. These studies aim to evaluate the optimal dosage, combination strategies, and patient selection criteria for Atovaquone-based therapies. Preliminary results from these studies have shown promising outcomes, warranting further investigation and clinical trials. Atovaquone, an anti-malarial drug, has emerged as a well-studied molecule with a high safety profile; its potential as an anticancer agent has been supported by pharmacological preclinical evidence demonstrating its ability to inhibit cancer cell growth and induce apoptosis [304, 306]. Ongoing research and clinical studies will shed more light on the efficacy and safety of Atovaquone in cancer therapy, paving the way for its potential inclusion as a repurposed drug in the treatment armamentarium against cancer.

### Myorelaxant agents

#### Thiocolchicoside

Thiocolchicoside is a chemically synthesized colchicoside produced from *Gloriosa superba* (Liliaceae) that has been authorized in Europe (EMA) only as an add-on treatment for painful muscle contractures (permanent tightening of the muscle tissue) [307]. Surprisingly, thiocolchicoside has lately been mentioned in several publications to have anticancer effects. Reuter et al., for instance, found that thiocolchicoside has anticancer effects in different malignant diseases including leukemia, lymphoma, and squamous cell carcinoma. Thiocolchicoside inhibited NF-B and COX-2 stimulation by promoting ubiquitination deterioration of IB, a major suppressor of the NF-B signalling cascade controlling IKK status and p65 nuclear translocation [308]. It seems that thiocolchicoside can block the receptor stimulator of the NF-kB ligand and the NF-B signalling cascade, which inhibits cancer-induced bone metastasis [309]. Different pharmaceutical firms market thiocolchicoside as a myorelaxant with anti-inflammatory and analgesic effects and advertise its use as a nociceptive medication [310]. The effects of thiocolchicoside, when used in the treatment of lower back pain, have been validated in several clinical trials [311, 312]. Despite the limited clinical trials exploring its anticancer effects, thiocolchicoside, a half-century-old medication, might be useful in cancer treatment through drug repurposing. However, this has to be proven in clinical trials, because until now no such evidence does exist.

## Conclusion

Human cancers are different diseases and, therefore, need different treatment approaches and options. There has been a significant development in discovering new drugs for different types of malignant diseases during the last two decades. However, due to acquired resistance to existing medicines, a considerable number of cancer patients are incurable, which causes frustration for scientists and physicians. The budgets of many countries for treating human cancers are limited, and they cannot afford the current chemotherapy treatments which are more and more expensive. As a result, drug repurposing has been identified as one of the most promising ways to find novel anticancer therapies that are cheaper and can be faster to obtain marketing authorization. Academics, scientists, and pharmaceutical businesses all recognize the value of drug repurposing in dealing with the rising burden of human cancers. Different types of drugs that can have anti-cancer cell effects have been discussed in this review. By targeting the well-studied mechanisms implicated in carcinogenesis, these drugs have been shown to limit cellular growth, metastasis, and invasion or induce cell cycle arrest and apoptosis. Clinical trials are currently being performed with repurposed drugs based on their preclinical anti-cancer efficacy. Some of them have been approved by the FDA for the treatment of human malignant diseases, such as raloxifene which has been approved for breast cancer, and thalidomide, which is used to treat multiple myeloma. A meta-analysis of medications including metformin, statins, and aspirin demonstrated their association with a lower risk of cancer, and these treatments may be licensed for cancer treatment in the near future. Scientists can now predict a treatment's efficacy, mode of action, and safety in different diseases, including cancer, thanks to advances in pharmacogenomics and high-throughput drug screening methods. Drug repurposing brings up a whole new world of research into existing drugs, potentially allowing more prompt and cheaper therapy for malignant diseases.

In conclusion, this comprehensive review explores the repurposing of non-oncology drugs for cancer therapy, focusing on elucidating mechanisms, evaluating efficacy, and exploring clinical prospects. One notable finding from our analysis is the observation of higher IC50 values associated with the repurposed compounds during in vitro studies. The higher IC50 values suggest that the repurposed compounds may exhibit reduced potency in inhibiting cancer cell growth compared to traditional anticancer agents. While this finding poses challenges, it also presents opportunities for future investigations. Understanding the underlying reasons for these elevated IC50 values is crucial for optimizing the therapeutic potential of repurposed compounds in cancer treatment. Several factors could contribute to the observed higher IC_50_ values, including differences in target specificity, altered pharmacokinetics, or complex interactions within the cancer microenvironment. Addressing these issues requires a multi-faceted approach involving in-depth mechanistic studies, refinement of drug formulations, and innovative combination strategies. To overcome the limitations posed by higher IC_50_ values, further research should focus on enhancing the effectiveness of repurposed compounds through various strategies. These may include identifying synergistic drug combinations, optimizing drug delivery systems to enhance bioavailability, or exploring novel formulations to improve target specificity. In addition, the use of advanced preclinical models that better recapitulate the complexities of human cancer biology could provide valuable insights into the efficacy of repurposed compounds. While higher IC_50_ values observed in vitro pose challenges, it is important to consider that repurposed drugs have the advantage of established safety profiles, known pharmacokinetics, and potentially reduced development timelines. By addressing the issue of higher IC_50_ values and leveraging the strengths of repurposed compounds, we can advance the field of cancer therapy by potentially identifying new treatment options that are both effective and safe. In summary, the observation of higher IC_50_ values for repurposed compounds during in vitro studies highlights the need for further investigation and optimization. By addressing these challenges head-on and capitalizing on the unique opportunities provided by repurposed drugs, we can accelerate the development of innovative cancer therapies and improve patient outcomes. This discrepancy raises important considerations for further research and clinical translation.

## Data Availability

Not applicable.

## Abbreviations

Akt: Protein kinase B
Bax: Bcl-2-associated X protein
Bcl2: B-cell lymphoma 2
BSE: Bovine spongiform encephalopathy
c-myc: Cellular myelocytomatosis
COX-2: Cyclooxygenase-2
CSCs: Cancer stem cells
CSCs: Cancer stem cells
CTGF: Connective tissue growth factor
DAPK: Death-associated protein kinase
DHFR: Dihydrofolate reductase
EGFR: Epidermal growth factor receptor
eIF4E: Eukaryotic translation initiation factor 4E
ER: Endoplasmic reticulum
FACT: Facilitates chromatin transcription
FDA: Food and drug administration
HDACs: Histone deacetylase
HER2: Human epidermal growth factor receptor-2
HIV: Human immunodeficiency virus
HUVEC: Human vascular endothelial cells
MCF-7: Michigan Cancer Foundation-7 cell line
MDA-MB-231: MD Anderson-Metastatic Brest-231 cell line
MMPs: Matrix metalloproteinases
mTOR: Mammalian target of rapamycin
MZ: Mebendazole
NF-Kb: Nuclear factor-kB
NIH: National Institute of Health
NO: Nitric oxide
NSAID: Non-steroidal anti-inflammatory drug
P53: Tumor protein TP53
PAK-1: Protein-activated kinases-1
PARP: Poly (ADP-ribose) polymerase
PCNA: Proliferating cell nuclear antigen
PSADT: PSA doubling time
PTB: Phosphotyrosine binding
ROS: Reactive oxygen species
Shc-PTB: Shc-Phosphotyrosine binding
SNEA: Sub-network enrichment analysis
SOD2: Superoxide dismutase 2
STAT3: Signal transducer and activator of transcription-3
TfR1: Transferrin receptor protein-1
TKIs: Tyrosine kinase inhibitors
TRAIL: TNF-related apoptosis-inducing ligand
VEGF: Vascular endothelial growth factor
XIAP: X-linked blocker of apoptosis
YAP1: Yes-associated protein-1
